# Combining value of information analysis and ethical argumentation in decisions on participation of vulnerable patients in clinical research

**DOI:** 10.1186/s12910-018-0245-x

**Published:** 2018-02-05

**Authors:** Gert J. van der Wilt, Janneke P. C. Grutters, Angela H. E. M. Maas, Herbert J. A. Rolden

**Affiliations:** 1 Department of Health Evidence (133), Radboud University Medical Centre, PO Box 9101, 6500HB Nijmegen, The Netherlands; 2 Department of Cardiology (616), Radboud University Medical Centre, PO Box 9101, 6500HB Nijmegen, The Netherlands; 3 Council for Public Health and Society, PO Box 194042500, CK The Hague, The Netherlands

**Keywords:** Vulnerable patients, Clinical trials, Specifying norms, Novel drugs, Value of information analysis, Clinical uncertainty

## Abstract

**Background:**

The participation of vulnerable patients in clinical research poses apparent ethical dilemmas. Depending on the nature of the vulnerability, their participation may challenge the ethical principles of autonomy, non-maleficence, or justice. On the other hand, non-participation may preclude the building of a knowledge base that is a prerequisite for defining the optimal clinical management of vulnerable patients. Such clinical uncertainty may also incur substantial economic costs.

**Main text:**

We present the participation of pre-menopausal women with atrial fibrillation in trials of novel oral anticoagulant drugs as a case study. Due to their non-participation in pivotal trials, it is uncertain whether for them, the risks that are associated with these drugs are outweighed by the advantages compared with conventional treatment. We addressed the question whether research of this new class of drugs in this subgroup would be appropriate from both, an ethical as well an economic perspective. We used the method of specifying norms as a wider framework to resolve the apparent ethical dilemma, while incorporating the question whether research of oral anticoagulants in premenopausal women with atrial fibrillation can be justified on economic grounds. For the latter, the results of a value-of-information analysis were used.

**Conclusions:**

Further clinical research on NOACs in premenopausal women with atrial fibrillation can be justified on both, ethical and economic grounds. Addressing apparent ethical dilemmas by invoking a method such as specifying norms can improve the quality of public practical reasoning. As such, the method should also prove valuable to committees that have formally been granted the authority to review trial protocols and proposals for scientific research.

## Background

The ethics of clinical research depends on the fulfillment of requirements that result from values such as respect for autonomy and welfare of research subjects, non-maleficence, and optimal use of scarce resources [1]. In concrete situations, such requirements can be mutually conflicting. For instance, subjects who are eligible based on the scientific objectives of a study, but who are at substantially higher risk of being harmed or experiencing more severe harm, should be excluded from participation on the basis of the risk-benefit requirement. If, however, the treatment under investigation is likely to be used in such patients, they should be included to learn how the treatment affects them, thereby enhancing the social value of the study ([1]; p. 2704). The method of specifying norms has been developed as a means to resolve such conflicts in a transparent and systematic way [2–4]. According to this method, establishing what follows from abstract, general values in concrete situations requires specification of those values. Specification is an argumentative process, where general norms are qualified by “adding clauses indicating what, where, when, why, by what means, by whom, or to whom an action is to be, is not to be, or may be done” so as to arrive at a more concrete interpretation about how best to honor the commitment to the general norm ([2]; p 295). Specifications are not fixed but not arbitrary either, and depend on the particular context of the case and the range of values that are taken into account [2]. When requirements do conflict, this does not result by necessity from the concerning values, but from the particular way these values were specified. The task, then, is to identify those values and to develop alternative or further specifications of those values that are plausible and acceptable, given the particularities of the case, the range of values that are taken into account, and the formal criteria of specification [2]. In this paper, the method of specifying norms will be applied to the issue of participation of vulnerable patients in clinical research. It has been argued that vulnerable patients deserve special protection in the context of clinical research, and that in many cases, vulnerability may be a reason why participation of such patients in clinical research is morally problematic [5–8]. This is also reflected in the various guidelines that have been released on the issue.^1^ At the same time, however, it has been pointed out that the reasons why patients are vulnerable may differ, and that this might affect our judgment of the propriety of their participation in clinical research. Specifically, a distinction has been made between consent-based vulnerability, risk-based vulnerability and justice-based vulnerability [9]. Consent-based vulnerability refers to situations where patients lack the capacity to go through the process of informed consent in the way we would like them to do. Examples are young children, and patients with advanced Alzheimer’s disease. Risk-based vulnerability refers to situations where patients have certain characteristics which put them at greater risk of sustaining harm from procedures that are being tested - for example due to relevant co-morbidity. Justice-based vulnerability refers to situations where participation in clinical research is unlikely to be of future benefit to patients who are in similar situations as the ones who are invited to participate. An example is studies that are being conducted in low and middle income countries that are unlikely to gain access to the treatments that are being tested, should they prove beneficial.

The current paper provides an example of the second class (risk-based vulnerability). It addresses the question whether premenopausal women with atrial fibrillation (AF) should participate in clinical studies of novel oral anticoagulants (NOACs). By this, we do not wish to suggest that women, generally, should be considered vulnerable. In the context of the use of NOACs, however, premenopausal women can be considered vulnerable, as explained below. AF is a type of arrhythmia, leading to the loss of effective contraction of the heart. The condition is known to increase the risk of ischemic stroke and other types of thromboembolic events, which is the rationale for anticoagulation in these patients [10]. Vitamin K antagonists (VKAs) have been the mainstay of oral anticoagulation for decades [11]. However, these drugs are considered cumbersome to use because of interactions with food and drugs and because of the requirement of frequent laboratory testing. This is deemed to be a key reason for poor compliance and inadequate anticoagulation [12]. Several attempts have been made to develop safe and effective alternatives to VKAs. Of these, dabigatran was the first NOAC to appear on the market, and was associated with rates of ischemic stroke, systemic embolism, and major hemorrhage that were similar to those associated with VKAs [13]. Apixaban, rivaroxaban and edoxaban quickly followed dabigatran as successful alternatives to VKAs [14–16]. The phase III trials on these four NOACs were conducted in patients with AF, aged 70–73 years, of which 60 to 65% were male. This in spite of the fact that 60% of people with AF are women and that women may sustain a higher risk of bleeding from anticoagulation [17]. This reflects the more generic phenomenon that women are still underrepresented in many cardiovascular clinical trials, while important gender differences are present within most areas of heart disease [18].

The number of premenopausal women using oral anticoagulation is increasing [19]. In a recent study among patients with AF in daily care, dabigatran, relative to warfarin, was associated with a higher risk of gastrointestinal and vaginal bleedings, but a lower risk of intracranial bleedings [20]. Evidence suggests that among premenopausal women, the risk of abnormal vaginal bleeding is higher in case of dabigatran as compared to warfarin [19]. In a small trial, it was found that rivaroxaban is associated with twice as many abnormal uterine bleeds as VKAs [21]. The issue is of particular significance, since effective reversal strategies to stop the bleeding exist for warfarin, but not for NOACs [22].

In sum, trial-based evidence exists, suggesting that NOACs provide similar protection to elderly patients with AF from thromboembolic events without incurring greater risk of bleeding. These NOACs do not have the practical disadvantages associated with conventional treatment. At the same time, NOACs are being prescribed in premenopausal women with AF, and evidence is accumulating that in these patients, NOACs, as compared to conventional treatment, are associated with greater risk of gastrointestinal and abnormal uterine bleeding. Since no established means exist to stop such bleedings, it is questionable whether in this group of patients the improved ease of use of NOACs outweighs the associated risks. Moreover, the number of premenopausal women with an indication for the use of NOACs for AF and other indications is steadily increasing.

The objective of our paper is to address two questions: 1) Was it right to exclude premenopausal women with AF from trial participation in the first place? 2) Now that premenopausal women with AF were in fact excluded from these studies, would further research on NOACs in these women be warranted, and if so, what type of research would be appropriate? To address these questions, the method of specifying norms was used, in conjunction with the results of a Value of Information (VoI) analysis. While the former is a formal model of moral argumentation, the latter provides a framework for incorporating the economic costs and benefits of conducting research aimed at reducing uncertainty. It can be considered as a means to inform the judgment whether a proposed clinical study represents social value, as defined by Emanuel et al. [1]. The two methods are different approaches that can be used to assess for concrete situations whether specific research is appropriate from a moral and from an economic perspective, respectively. We will argue that the results of the VoI analysis can be incorporated in the wider framework, offered by the method of specifying norms.

## Main text

### Specifying norms

Specifying norms is an established method of moral argumentation [23]. It has been used to address issues ranging from the management of serious incidental findings in brain imaging research when consent for disclosure is declined [24], to global development [25]. In the following, we will illustrate the method of specifying norms, using an abbreviated form of an example presented by Richardson ([2], pp. 303–305). The case consists of a severely malformed newborn child, whose parents have indicated that it is their sincere wish that the child should be allowed to die by withholding nutrition and hydration. The first step in the moral inquiry is to acknowledge that the case presents an apparent moral dilemma. In the literature, this has also been referred to as experiencing moral perplexity: being uncertain as to what constitutes the morally right thing to do or how to proceed in specific cases [26].^2^ The next step in the inquiry consists of identifying the moral commitments that seem to cause the uncertainty. Richardson proposes three moral norms that are relevant to this particular case and worthy of our commitment:

1. Generally speaking, it is wrong to kill innocent persons.
2. Generally speaking, we should respect the reasonable choices of parents regarding their children (respecting parental autonomy).
3. Generally speaking, we should act in the best interest of persons who have been entrusted to our (professional) care.

The third step in the inquiry is to realize that there is always a gap between general moral norms (such as respecting autonomy) and judgments as to what follows from our commitment to such norm in concrete situations.^3^ An attractive way to bridge this gap is by making the norm progressively more specific. There are multiple ways of specifying a norm in a concrete situation, which will depend on the specific context, but also and crucially on the range of norms that are jointly taken into consideration. The challenge is to develop specifications that epitomize maximal coherence among our moral commitments and between our commitments and the decisions and actions we (propose to) take. In the example, Richardson suggests that the inquiry might proceed as follows:

Deliberators might come to realize that the first norm (wrong to kill innocent persons) can in fact be conceived as a specification of a still more general norm, expressing respect for persons. This could prompt the question ‘What is it about persons that make them worthy of our respect?’.^4^ An answer to that question could be: their self-consciousness. This could lead to a re-specification of the first norm, reading: “It is generally wrong to kill innocent people *who have attained self-consciousness or who have the potential to develop self-consciousness over time*.” If it can be demonstrated (drawing on relevant empirical evidence) that this is extremely unlikely in the current case, our commitment to this norm would no longer prohibit us from letting the child die. It would resolve the dilemma, in the sense that it leads to a practical recommendation that is consistent with the various moral commitments that were identified. It is important to note that if we were to take part in the deliberation and bear responsibility for the decision, we would not be asked to relinquish our commitment to the relevant general norms. What *would* be required from us, is to develop and consider multiple specifications of those commitments in a way that is relevant to the case at hand and, in doing so, strive for maximal coherence. Thus, the process is characterised by an element of continuity, or stability, as well as an element of flexibility. The process of specifying norms resists formalisation, and the outcome is not fixed from the outset. In the example, for instance, if there would be the prospect of some level of self-conscious life (an empirical issue), the second norm could be re-specified to read: “Generally speaking, we should respect the reasonable choices of parents regarding their children *so long as they respect the children’s rights*.” Defying the parents’ choices in this particular case on those grounds would, then, be consistent with the requirements of the first norm. The example shows the crucial role of facts in the deliberation, but also that the relevance of those facts depends on the norms that are brought to bear on the case. An important requirement of the argumentation is that the normative force of the general norms is still captured in the specified versions. Arguably, acceptance of proposed re-specifications of norms is something that needs to be judged by those who are directly involved. Thus, the argumentative model is formal, requiring the input from those who are willing to join the deliberation. The model can be considered to help structuring the deliberative process.

## Value of information analysis

Value of Information (VoI) analysis is a method for assisting decision making on the allocation of resources to scientific research [27, 28]. The general idea is that conducting research costs money, and that such money can be used for other purposes as well. Hence, spending money on research entails ‘opportunity costs’: benefits that would result from alternative modes of spending the money. Scientific research can be used to reduce uncertainty on a particular issue, and uncertainty, too, can incur costs. When we are forced to make decisions under uncertainty, there is a probability of making the wrong decision with its associated (societal) costs. Collecting information through scientific research can, to some extent, reduce that probability.

An example from the context of medical treatment is the cesarean section. One may, for example question in which cases this surgical procedure is appropriate. When there is substantial uncertainty on this issue, both over- and under-treatment are likely to occur, incurring substantial material and immaterial costs. Further research may shed more light on the question in which cases a cesarean section is likely to be beneficial to the mother and the child. Conducting an appropriate empirical study would be a sensible attempt to clarify the issue. Such a study would cost money, but so does incorrectly (not) conducting a cesarean section. The VoI analysis provides a formal model to estimate and monetarise outcomes of (not) conducting specific research. It puts the various costs and benefits into a single, unifying analytical framework, informing the decision whether such research would be a wise way of spending a community’s resources.

## Case study

We will now turn to the two central questions of our paper, the first of which being:

Q1: Should premenopausal women with AF have been included in trials of NOACs in the first place?

The method of specifying norms urges us to ask whether in answering this question we must confront a moral dilemma, and, if so, what moral commitments seem to be responsible for the dilemma. In response to this question, we suggest the following: at the time when the trials were conducted, a reason for excluding premenopausal women with AF from trials of NOACs may have been that there was sufficient reason (although no evidence) to believe that these women were at greater risk (as compared to male patients or postmenopausal women with AF) of sustaining (serious) side-effects. If true, this would adversely affect their benefit-risk ratio: the potential of harm resulting from trial participation might not be outweighed by the potential for benefit. Another reason for excluding them from such trial might have been that they would have ‘diluted’ the trial: with a more heterogeneous trial population it would have been more difficult to demonstrate an added value of NOACs as compared to conventional anti-coagulation. This would have required a larger trial population, leading to higher costs and presumably longer accrual period.

In contrast, a reason to include premenopausal women with AF in the phase III trial on NOACs is that this could have resulted in an evidential basis to guide the future clinical management of these patients. Tentatively, these considerations could be seen as specifications of the following norms:

1. Generally speaking, for prospective participants, the overall benefit associated with trial participation should outweigh the overall harm.
2. Generally speaking, treatments that are potentially of added value should be made available to patients as quickly as possible.
3. Generally speaking, treatments should not be offered to patients (as opposed to study participants) unless there is sufficient evidence that they are effective and safe.

The third norm basically implies that, in the *current* situation, premenopausal women with AF need to accept that, even though there is an indication for anticoagulation, they do not qualify for NOACs (which do not entail the need for regular laboratory testing), because there is reason to believe (although no hard evidence) that these substances might lead to more (serious) bleedings that are difficult to control. The norm may be considered to be a specification of the more general norm ‘In dubio abstine’: in case of doubt, do not proceed by using an intervention of which you are not sure that it will do the patient good and not harm her [29]. If we find this hard to accept, it should urge us to also include such ‘vulnerable’ patients in future trials. Can this, then, be made coherent with the other two considerations? With respect to the second norm, we would suggest that this is an important consideration, but particularly in case of patients suffering from a disease for which there is currently no effective treatment. This is also the essence of the novel policy of the European Medicines Agency to ensure early access to medicines through adaptive pathways [30]. The second norm might be specified, then, by stipulating that this holds in case of unmet medical needs only.

The first norm seems to express the notion that it would be unreasonable, or perhaps even unfair, to expect from premenopausal women with AF to participate in trials of NOACs [1]. For, the most likely outcome would be that this would reveal that indeed, NOACs cause more, and more serious bleedings in these patients as compared to other patients with AF. As such, it would produce the evidential basis for the clinical management of future premenopausal women with AF. Not only does trial participation entail no benefit for participants themselves, it would also put them at risk of serious adverse events. At this point of the deliberation, we might ask, however, whether this would not be unduly paternalistic [31]. Perhaps, patients should be allowed to make an informed choice in this matter. If conventional treatment is, in fact, experienced as quite cumbersome by a patient, and she is willing to accept the risk of bleeding, should she then not be offered the choice to participate in the trial? Indeed, patients might be willing to accept the risk, *and* decide that they would like to contribute to the further production of relevant evidence that can serve to guide future clinical management. This could be considered as a choice that is at least partially altruistically informed, and we should perhaps not deprive patients from making such choices [32]. What happens here, in the argument, is that yet another norm has come into view, to wit, the idea that we cannot completely rule out that persons sometimes wish to behave from motives that are at least partially altruistic, and that we should not prevent that from happening. It is up to the deliberators to decide whether they wish to espouse this idea, whether they consider it relevant to the inquiry, and how it might affect the specification of the first norm. For instance, the first norm could be re-specified, reading ‘Generally speaking, for prospective participants *who are not altruistically motivated*, the overall benefit associated with trial participation should outweigh the overall harm’. If deliberators would agree that this is a reasonable way to achieve greater coherence in their moral commitments through specifying and re-specifying the various norms, this would suggest that inclusion of premenopausal women with AF in trials of NOACs would, in fact, have been the preferred option.

Q2: Now that premenopausal women with AF have been excluded from trials of NOACs, is further research warranted?

We know that reality has taken a different course. As a consequence, the question whether NOACs can be considered a safe treatment for premenopausal women with AF is as yet unresolved. Since we have tried to argue that those patients should have been included in those trials in the first place, there seems to be no good reason why such a trial should not be warranted now. However, the situation is somewhat different and an additional question that might be asked is whether further research in this area would constitute value for money. After all, substantial resources have been spent on clinical research of NOACs, and other areas of clinical research may deserve higher priority. The question is particularly relevant, given the recent reports in the literature that there is an urgent need to increase value and reduce waste in biomedical research [33]. Clearly, this introduces yet another moral concern. If we are willing to commit ourselves to this norm, further empirical evidence is needed to answer the question whether conducting research in this area would be consistent with the objective of increasing value and reducing waste in biomedical research.

One way of answering this question is by conducting a VoI analysis. When applied to the case of NOACs in premenopausal women with AF, such analysis reveals that on the basis of the currently available evidence, there is a 22% probability that for these patients, VKAs is the preferred option [34]. It is estimated that this uncertainty would translate into a monetary value of approximately 22 million Euros. Since the costs of gathering further empirical evidence on this issue are likely to be substantially lower, further research does seem to be warranted, also from an economic point of view. Since the key question seems to be whether NOACs are safe for this subgroup of patients, rather than whether they are effective, an observational study may be the most appropriate design [35]. Of course, the same conditions hold that were suggested above, of the risks being controllable and, upon due information, acceptable to patients. In some settings, existing systems for pharmacovigilance may suffice, whereas in other settings it might be necessary to set up specific registries.

## Conclusions

We have tried to argue that premenopausal women with AF could be considered a specific type of vulnerable patients, since they are at greater risk than other patients with AF of sustaining abnormal, difficult to control bleedings when exposed to NOACs. As such, the question whether they should be allowed to participate in trials of those drugs needs careful consideration. Using the method of specifying norms, we have argued that the participation of these patients, under certain conditions, is morally defensible, indeed morally preferable to their exclusion from those trials. We have also tried to show that an economic analysis such as a VoI can be incorporated in such argument. It provides estimates of empirical data that are relevant, once we have espoused a moral norm such as the increase of value and the reduction of waste in biomedical research [1]. Our conclusion can be considered as a specific example of including vulnerable patients in pragmatic trials, a conclusion that was also recently advocated by Welch et al. [36].

Various approaches have been developed to help resolve ethical dilemmas in a systematic and transparent way, including specifying norms, casuistry, wide reflective equilibrium, and balancing. A comparison between these approaches is well beyond the scope of this paper. Suffice to conclude, perhaps, by stating that presumably the most valuable aspect of a method such as specifying norms lies in relating our daily behavior to norms that we have reason to value. In using the method, these norms acquire practical significance. At the same time, the moral significance of our daily behavior is revealed. It does so in a way that recognizes and tries to preserve the non-determinate character of our daily affairs and our interpretations thereof. It does so in a way that accepts that moral dilemmas are a fact of life, and that it is rational to seek maximal coherence in our moral commitments. And it does so in a way that is transparent and that can be shared. As such, it should also prove valuable to committees that have formally been granted the authority to review trial protocols and proposals for scientific research.

## Abbreviations

AF: Atrial fibrillation
NOACs: Novel oral anticoagulants
VKAs: Vitamin K antagonists
VoI: Value of Information

## Footnotes

1 e.g., the Declaration of Helsinki of the World Medical Association, the International Ethical Guidelines for Biomedical Research Involving Human Subjects from the Council for International Organizations of Medical Sciences, and the report on Ethical and Policy Issues in Research Involving Human Participants from the National Bioethics Advisory Commission.

2 Such uncertainty may reside within one person, or may arise from conflicting understandings among persons (‘conflicting certainties’).

3 This part of a moral argument has also been referred to as explication of moral norms, which provides an answer to the question: what follows from our commitment to this norm in this specific situation? [26]

4 This part of a moral argument has also been referred to as the rationale of moral norms, which provides an answer to the question: why should we be committed to this norm in the first place? [26]
